# BREAST CANCER, DERMATOFIBROMAS AND ARSENIC

**DOI:** 10.4103/0019-5154.48981

**Published:** 2009

**Authors:** Paul I Dantzig

**Affiliations:** *From the Department of Dermatology, Columbia University School of Medicine, New York, USA*

**Keywords:** *Arsenic*, *breast cancer*, *dermatofibromas*

## Abstract

**Background:**

Dermatofibromas are common benign tumors in women, and breast cancer is the most common malignancy in women. The aim of this study is to determine if there is any relationship between the two conditions.

**Materials and Methods:**

Five patients with dermatofibromas and 10 control patients (two groups) had their skin biopsies measured for arsenic by inductively coupled mass spectrometry. Fifty randomly selected patients with breast cancer and 50 control patients were examined for the presence of dermatofibromas.

**Results:**

The dermatofibromas were found to have an arsenic concentration of 0.171 micrograms/gram, compared with 0.06 and 0.07 micrograms/gram of the two control groups. Forty-three out of 50 patients with breast cancer had dermatofibromas and 32/50 patients with breast cancer had multiple dermatofibromas, compared to 10/50 control patients with dermatofibromas and only 1/50 with multiple dermatofibromas.

**Conclusions:**

Arsenic is important in the development of dermatofibromas and dermatofibromas represent a reservoir and important sign of chronic arsenic exposure. Dermatofibromas represent an important sign for women at risk for breast cancer, and arsenic may represent the cause of the majority of cases of breast cancer.

## Introduction

Breast cancer is the most common malignancy and the second leading cause of cancer deaths in women. Its incidence has been steadily increasing over the last 50 years, with a small decline noted in the period 2000-2005, due to a reduction in hormone replacement therapy.[1] Numerous studies have been done on the etiology of breast cancer, but no carcinogens have been identified to this date, even though some risk factors have been determined. No physical signs have ever been identified to show who is at risk for developing breast cancer.

Dermatofibromas are common benign tumors which are more common in women. They usually develop in people in their 20's and 30's and are most common on the legs though any part of the body can be affected. Their cause is unknown though minor trauma may precipitate their development. A few cases have been associated with immune problems.[2]

## Materials and Methods

Five patients with clinical dermatofibromas were randomly selected for the study. There were four women and one man, and their ages ranged from 39 to 54. Three of the women had a history of breast cancer, and three of the patients had a history of squamous cell carcinoma of the skin. Informed consent was obtained from each patient prior to examination and biopsy. An excisional or deep shave biopsy was performed on each lesion. Each biopsy was bisected, with half sent for routine histopathologic exam and the other half evaluated for arsenic by inductively coupled mass spectrometry.[3] A blood arsenic level was obtained at the time of the biopsy to rule out any recent exposure to arsenic. As a control, the specimens were also examined for mercury, a metal which is frequently found in food as a pollutant with arsenic and which can also be found in the skin.[4,5]

Two control groups were randomly selected for the first phase of the study. The first group consisted of five patients with no detectable blood arsenic, who underwent a deep skin biopsy. This included one squamous cell carcinoma, one cyst, one intradermal nevus, one dysplastic nevus and one plaque of lichen planus. The second group consisted of five patients with detectable blood arsenic (5 micrograms/liter-9 micrograms/liter) and included one keloid, one intradermal nevus, one basal cell carcinoma, one verruca vulgaris and one seborrheic keratosis. Fifty patients with breast cancer were randomly selected for the second phase of the study through radio and newspaper advertisements. Their ages ranged from 39 to 65, with a mean age of 46 years. Patients over the age of 70 were excluded from the study because dermatofibromas can regress with age; and, the changes in the skin with age, including laxity, hyper and hypopigmentation, solar damage and the development of keratosis, could make the evaluation and diagnosis of any lesion difficult and they lead to false negative results. A control group of 50 women ranging in age from 37 to 65, with a mean age of 55 years, without breast cancer, was randomly selected for this study.

After receiving informed consent, the patients were questioned about their breast cancer, including the age at which it occurred, the type of tumor, family history and the presence of breast cancer (BRCA) genes. The patients then received a total body skin exam to identify any potential dermatofibromas and if any were found, they were questioned about their duration. The dermatofibromas were diagnosed clinically by a firm, movable, non painful dermal or elevated or flat papule, which had a dusky red or mottled pigmented appearance and a rim of hyperpigmentation and may present with a dimple on compression. No biopsies were performed.

## Results

The five patients with dermatofibromas had their tumors confirmed by histopathology, and none of the patients had measurable blood arsenic at the time of their biopsies. Analysis of the tumors by inductively coupled mass spectrometry showed an arsenic level of 0.171 micrograms/gram. Due to the inclusion of some normal skin with the tumor, this value may actually be significantly higher. The control group with detectable blood arsenic showed 0.06 micrograms/gram of arsenic, and the control group without detectable blood arsenic showed 0.07 micrograms/gram of arsenic.

Of the 50 patients with breast cancer, 43 had dermatofibromas and 32 had multiple dermatofibromas [Table 1]. When patients were asked how long they had had the lesions, none could give an exact time, but they all stated that they had the lesions years before the breast cancer. Most stated that they thought they began after minor trauma. Even though seven patients did not have dermatofibromas, their absence does not preclude exposure to arsenic because dermatofibromas sometimes regress with age, the lesions could have been missed by the physician or the patient may not have experienced the necessary trauma to the endothelial cells, which is postulated to precipitate the lesions.

**Table 1:** Details of patients and controls

	No dermato fibromas	One dermato fibroma	Multiple dermato fibroma
Control group	40/50	9/50	1/50
Breast cancer group	7/50	11/50	32/50
Control group with strong family history of breast cancer	5/5	0/5	0/5

Of the 50 patients without breast cancer, nine had a single dermatofibroma, and one had multiple dermatofibromas. Though measurements were not taken, they all appeared noticeably smaller than in the breast cancer group. Five of the patients without breast cancer and without dermatofibromas had positive family histories (mother and sister) of breast cancer, but none had BRCA genes. Though 10 of the 50 patients had dermatofibromas, a need for follow up for many years is still needed to see if they remain disease free and in the breast cancer free group.

## Discussion

The cause of dermatofibromas has remained an enigma even though the lesions are thought to be quite common. Since the exact incidence of dermatofibromas has never been studied, it is impossible to tell if there is any correlation with the rise in breast cancer. However, our control group shows that the incidence is approximately 20%, and the incidence of multiple dermatofibromas is less than 2%. While the initiating factor in dermatofibromas is unknown, trauma or damage to the superficial dermis is strongly suspected, and this is supported by the report of multiple dermatofibromas in patients with HIV and systemic lupus erythematosus in which damage to the endothelial cells occurs. In the history of patients, many state that the lesions develop after a minor cut, insect bite or shaving injury.[6–8]

The high concentration of arsenic in the dermatofibromas in the absence of arsenic in the blood suggests that it is a dynamic process in which small amounts of arsenic are being trapped in the tumor and that it acts as a reservoir for arsenic. This is important because the dermatofibroma exhibits two important characteristics of acute and chronic arsenic toxicity. First, there are several cellular variants of dermatofibromas, but, in most, endothelial cells are present in large numbers, suggesting an endothelial origin to the tumor.[9] Arsenic has an affinity for endothelial cells, which is the reason petechiae are a characteristic sign of acute arsenic poisoning and this toxic damage to endothelial cells could lead to reactive proliferation.[10] Secondly, chronic arsenic toxicity causes hyperpigmentation with hemosiderin and melanin, a feature common to dermatofibromas, after several months or years.[11] The finding of arsenic in dermatofibromas gives an important clue to chronic exposure to arsenic, a powerful carcinogen in humans, years before the breast cancer develops.[12]

Arsenic is a highly toxic and carcinogenic metal, which people are exposed to naturally in the environment and as a pollutant from industrial processes and chemicals. It has been used extensively as a pesticide on crops, at homes and pressure-treated wood, especially in its highly toxic trivalent and pentavalent forms. Arsenic is easily absorbed by plants and leaches in the soil and it easily enters the human food supply through water, seafood and fruits and vegetables. It has a half life of only 6-12 hours in the blood and concentrates in the kidneys and urine, which makes the finding of high concentrations in the dermatofibromas even more significant. Its exact mode as a carcinogen is unclear, but it is known to displace phosphorous in adenosine triphosphate (ATP), affecting DNA synthesis. It inhibits DNA ligation and repair, and it interferes with DNA methyltranferase, resulting in inactivation of tumor suppressor genes through DNA hypermethylation.[13,14]

Numerous studies have been done on breast cancer, in an attempt to link it to environmental agents, especially insecticides.[15] These studies were prompted by the very high incidence in areas like Long Island, NY, which have a history of extensive agricultural activities. The problem with these studies is that they need to study exposure for five, 10 or even 20 years prior to the development of the clinical cancer, when the DNA changes are occurring, and the dermatofibroma enables the physician to proceed back in time, to the period when the cell damage may have occurred. Numerous other studies have tried to link breast cancer to diet,[16] hormones,[17] genetics,[18] ethnicity,[19] obesity,[20] reproduction history,[21] childhood exposures,[22] and radiation[23] as causes or risk factors, yielding some important clues. Physical signs predisposing to the disease, however, have not been identified.

The finding of dermatofibromas, especially multiple dermatofibromas, in women with breast cancer provides an important and simple screening tool to identify women at risk, years before the clinical signs of the disease appear. The finding of arsenic in the dermatofibroma shows who is chronically exposed to this carcinogen and strongly suggests that arsenic maybe the cause of a majority of the cases of breast cancer. The finding that dermatofibromas may represent a reservoir for arsenic mandates that studies need to be done to see if other reservoirs may exist, especially in the breast, and to determine if these tumors may impede the rapid excretion of arsenic, making the body exposed to an inordinate amount of arsenic and increasing its carcinogenic potential. Since most arsenic enters the body through food, eliminating arsenic from water, fruits, vegetables (especially from contaminated farmland) fish, poultry, etc. should enable physicians and the society to dramatically reduce the number of cases of breast cancer in 10-20 years.
